# Atypical BSE (BASE) Transmitted from Asymptomatic Aging Cattle to a Primate

**DOI:** 10.1371/journal.pone.0003017

**Published:** 2008-08-20

**Authors:** Emmanuel E. Comoy, Cristina Casalone, Nathalie Lescoutra-Etchegaray, Gianluigi Zanusso, Sophie Freire, Dominique Marcé, Frédéric Auvré, Marie-Magdeleine Ruchoux, Sergio Ferrari, Salvatore Monaco, Nicole Salès, Maria Caramelli, Philippe Leboulch, Paul Brown, Corinne I. Lasmézas, Jean-Philippe Deslys

**Affiliations:** 1 Institute of Emerging Diseases and Innovative Therapies, CEA, Fontenay-aux-Roses, France; 2 Istituto Zooprofilattico Sperimentale del Piemonte, Turin, Italy; 3 Policlinico G.B. Rossi, Verona, Italy; 4 Scripps Florida, Jupiter, Florida, United States of America; 5 Genetics Division, Brigham & Women's Hospital, Harvard Medical School, Boston, Massachusetts, United States of America; University of Edinburgh, United Kingdom

## Abstract

**Background:**

Human variant Creutzfeldt-Jakob Disease (vCJD) results from foodborne transmission of prions from slaughtered cattle with classical Bovine Spongiform Encephalopathy (cBSE). Atypical forms of BSE, which remain mostly asymptomatic in aging cattle, were recently identified at slaughterhouses throughout Europe and North America, raising a question about human susceptibility to these new prion strains.

**Methodology/Principal Findings:**

Brain homogenates from cattle with classical BSE and atypical (BASE) infections were inoculated intracerebrally into cynomolgus monkeys (*Macacca fascicularis*), a non-human primate model previously demonstrated to be susceptible to the original strain of cBSE. The resulting diseases were compared in terms of clinical signs, histology and biochemistry of the abnormal prion protein (PrPres). The single monkey infected with BASE had a shorter survival, and a different clinical evolution, histopathology, and prion protein (PrPres) pattern than was observed for either classical BSE or vCJD-inoculated animals. Also, the biochemical signature of PrPres in the BASE-inoculated animal was found to have a higher proteinase K sensitivity of the octa-repeat region. We found the same biochemical signature in three of four human patients with sporadic CJD and an MM type 2 PrP genotype who lived in the same country as the infected bovine.

**Conclusion/Significance:**

Our results point to a possibly higher degree of pathogenicity of BASE than classical BSE in primates and also raise a question about a possible link to one uncommon subset of cases of apparently sporadic CJD. Thus, despite the waning epidemic of classical BSE, the occurrence of atypical strains should temper the urge to relax measures currently in place to protect public health from accidental contamination by BSE-contaminated products.

## Introduction

Classical Bovine Spongiform Encephalopathy (cBSE), the first prion disease identified in cattle, was initially reported in 1986 in the UK. Food-borne transmission of cBSE to humans was observed ten years later as a variant form of Creutzfeldt-Jakob Disease (vCJD) [1], leading to a major public health crisis.

This strain of cBSE is now rapidly disappearing as a result of appropriate containment measures. However, atypical forms of BSE have recently been identified in Europe and North America as a consequence of cBSE testing performed in these countries [2]–[4]. Because these cases are only found sporadically in older animals (≥8 years) coming to slaughter with few or no signs of disease, it would be plausible to suppose that atypical forms of BSE may have a lower virulence than cBSE and be innocuous to humans. However, recent studies suggest that one of the two main forms of atypical BSE, initially discovered in Italy and referred to as the bovine amyloidotic spongiform encephalopathy (BASE), might be at the origin of the cBSE epidemic: inoculation of the BASE strain into transgenic and inbred mice showed an apparent natural evolution towards the typical BSE strain [5], [6]. Moreover, a possible link has been suggested between BASE and one subtype (MV2) of human sporadic CJD (sCJD) on the basis of biochemical similarities [2], [7]. In contrast to vCJD, sCJD is believed to occur de novo without food-borne transmission. However, specific contaminating events by ingestion are difficult to rule out because human prion diseases can have silent incubation periods exceeding 50 years, as demonstrated for kuru [8].

One strategy to evaluate the risk of BASE for humans consists in assessing the susceptibility to disease transmission and the degree of pathogenicity in a non-human primate model that has already been shown to have characteristic clinical signs, histopathological lesions and PrPres profiles following infections with either BSE or vCJD [9], [10]. We therefore inoculated cynomolgus macaque monkeys (*Macacca fascicularis*) intracerebrally with BASE, cBSE and vCJD prion strains. The BASE strain, prepared from brain extract of a 15-year-old asymptomatic cow induced a distinctive and more rapidly fatal disease than cBSE, and showed a biochemical signature similar to that of the MM2 cortical subtype of human sCJD.

## Methods

### Cattle and human samples

The BASE inoculum (mix of brainstem and thalamus) from an asymptomatic 15 year-old Italian Piemontese cow [2]: 250 µl of a 10% brain homogenate in 5% glucose were inoculated intracerebrally (i.c.) to a single macaque monkey. As controls, we used two macaques inoculated i.c. with cBSE (brainstem from infected UK cattle) and 4 macaques inoculated i.c. with human vCJD [9], [11]. Twenty-one subjects with a diagnosis of definite sCJD were referred to the Medical Center in Verona, Italy during the period 2000–2004. Tissues were processed 4–18 hours post-mortem according to established guidelines regarding safety and ethics. Brains were cut longitudinally into two halves. Hemi-brains were frozen and stored at −80°C until biochemical studies were performed. The patient group encompassed all of the different Western blot subtypes of sCJD described by Parchi et al [7]: MM1 (5 cases), MV1 (2), VV1 (1), MM2 (4), MV2 (6) and VV2 (3).

### Non-human primate model

Cynomolgus macaques (*Macacca fascicularis*), captive-bred from the Centre de Recherche en Primatologie (Mauritius), were checked for the absence of common primate pathogens before importation and handling in accordance to national guidelines. Animals were maintained in biological security level 3 animal facilities and clinical examinations were performed regularly. They were humanely euthanized at the terminal stage of the disease, and tissues were either fixed in Carnoy's fluid for histological examination or snap-frozen in liquid nitrogen and stored at −80°C for biochemical analyses.

### Neuropathology and immunochemistry

Neuropathology and immunochemical detection of proteinase-resistant prion protein (PrPres) and Glial fibrillary acidic protein (GFAP) was performed on brain sections as previously described [12].

### PrPres analysis

Tissues were homogenized to 20% (w/v) final concentration in a 5% sterile glucose solution. PrPres was purified according to a protocol optimized for strain discrimination in ruminants [13], [14] (Discriminatory kit ref 3551177, BioRad, Marnes la Coquette, France). Briefly, brain homogenates were first subjected to proteolysis using either 0.4 µg (“low” concentration) or 4 µg (“high” concentration) of proteinase K/mg of brain (final concentration) in a special buffer that partially protects the N-terminal part of PrPres in order to increase strain discrimination, and then purified PrPres was concentrated by centrifugation. Purified, non-human primate and human samples were processed for Western blot analysis as previously described: briefly, samples were separated by electrophoresis on a 12% SDS polyacrylamide gel, blotted onto a nitrocellulose membrane and detected by two mouse monoclonal antibodies: the antibody from the BioRad Discriminatory kit, which targets the epitope WGQPHGGX within the N-Terminal octarepeat region at position 57–88, and 3F4, which targets the epitope MKHM in the hydrophobic core at position 109–112. The protein bands were visualized using a peroxidase-conjugated goat anti-mouse antibody and chemiluminescence.

## Results

### Transmission characteristics of BASE and BSE

#### Clinical features

The BASE-inoculated macaque developed clinical signs after a 21 months incubation period. Clinical signs evolved slowly during the first four months, being limited to mild tremor and myoclonus, without impairment in coordination or locomotion, and without anxiety or aggressiveness. In the last month, the clinical picture rapidly worsened with evidence of major spatial disorientation (the animal did not recognize its environment and seemed lost in its cage), cognitive troubles (no recall of food location and at intervals unaccountably stopped eating) and the appearance of incoordination and disequilibrium; however, appetite and general fitness were maintained. Euthanasia was performed at the terminal stage of illness at 26 months post inoculation (Table 1). The two cBSE-inoculated animals had longer incubations periods (37.5 months) and survivals (40 months) despite a presumably larger infecting dose (100 mg containing a 10-fold higher PrPres concentration). Moreover, the clinical presentation was very different: animals exhibited aggressiveness and anxiety in combination with incoordination, severe ataxic tremor, and loss of appetite to the point of near starvation. The four animals inoculated with human vCJD had a clinical evolution similar to that of animals inoculated with BSE, though with less prolonged survivals (25 to 37 months).

**Table 1:** Survival times of macaques inoculated intracerebrally with brain homogenates from cattle with BASE or BSE, and from humans with vCJD.

Strain	Source	Dose*	Survival time (months)
BASE	cattle	25 mg	26
BSE	cattle	100 mg	40
BSE	cattle	100 mg	40
vCJD	human	40 mg	25
vCJD	human	40 mg	30
vCJD	human	40 mg	32
vCJD	human	40 mg	37

* Amount of crude brain in 10% brain suspension inoculated intracerebrally. BSE brain had a 10-fold greater concentration of PrPres than the BASE brain). Animals inoculated with vCJD also received the equivalent of 8 mg of brain by intra-tonsillar injection.

#### Histopathology (Figures 1 and 2)

In the BASE-inoculated animal, the cortex showed widespread spongiosis and gliosis that were especially prominent in the fourth and fifth layers. Spongiosis was intense in the frontal cortex, with a loss of pyramidal cells in the third and fifth layers. Lesions in the parietal cortex were even more severe, with a complete disappearance of neurons in the fourth layer. In the cBSE-inoculated animals, spongiosis and gliosis were more discrete, and mainly affected the occipital cortex. In the obex and cerebellum, the lesions (spongiosis and loss of Purkinje and granular cells) were less pronounced in BASE than cBSE-infected animals.

**Figure 1:**
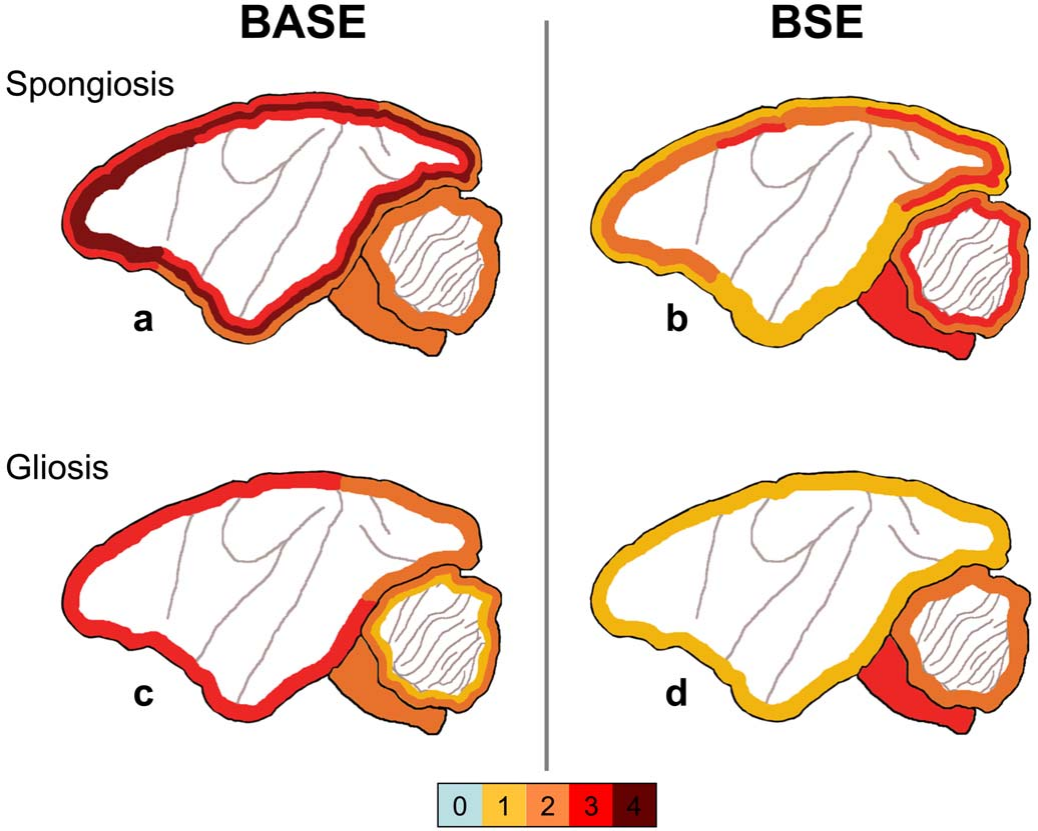
Diagrammatic representation of histologic lesions. Topographic distribution of spongiosis (a and b) or gliosis (c and d) in BASE and cBSE-infected primates. The lesions were scored from 0 to 4 (negative, light, mild, moderate, and severe).

**Figure 2:**
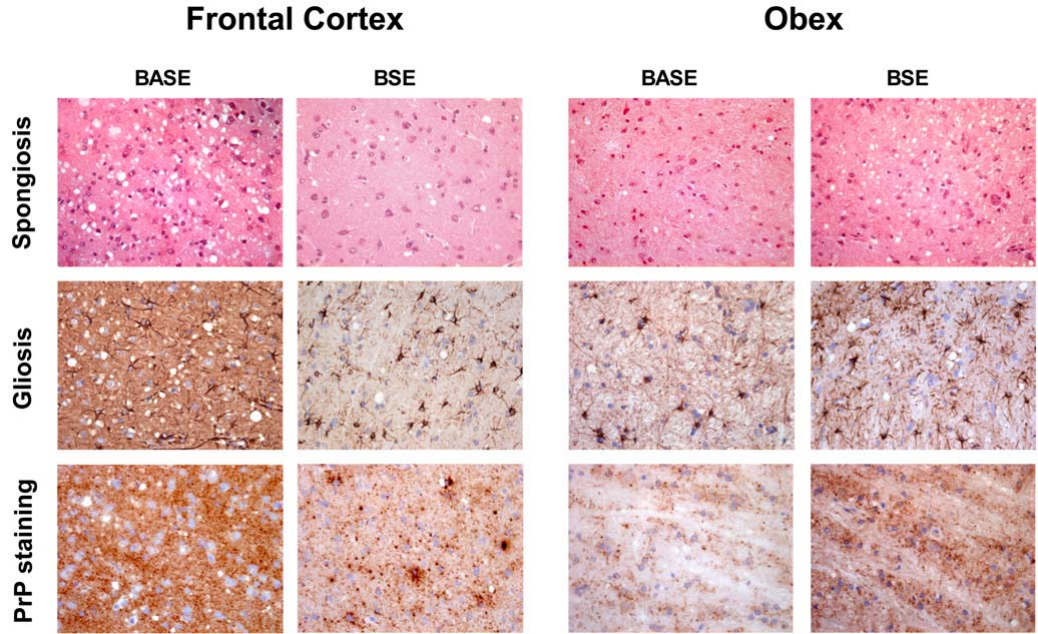
Histopathology and PrPres immunostaining. Spongiosis, gliosis (GFAP staining) and PrPres deposition in frontal cortex and obex in BASE- and cBSE-infected primates (original magnification ×200 for spongiosis and gliosis, ×400 for PrPres staining). Immunostaining of PrPres was performed with 3F4 monoclonal anti-PrP antibody after proteinase K treatment as previously described [11]. No staining was observed in the brain of control healthy primates (data not shown) in these conditions.

#### Immunohistochemistry (Figure 2)

In the BASE-infected animal, PrPres was distributed in a diffuse synaptic pattern (either fine and sandy or roughly granular) with laminar enhancement in the parietal cortex but no evidence of plaques, even when stained with thioflavine T (data not shown), whereas cBSE-infected animals had weak diffuse synaptic labeling but multiple intensely-stained PrPres aggregates and characteristic plaques [9].

### Strain discrimination by proteinase K sensitivity and antibody reactivity

We made use of a technique developed to discriminate and classify prion strains in small ruminants [14], based on the differential sensitivity of the octapeptide and core regions of PrPres proteins to proteinase K (PK) digestion. Controlled conditions of proteolysis allowed a strain-dependent threshold of removal of the octapeptides. This method, illustrated in Figure S1 (supplementary data), was successfully applied for the diagnosis of the first case of cBSE in a goat [15] and has now been validated by the European Commission for regular use on field. We adapted this test to primate prion strains, using only the higher PK concentration and substituting the monoclonal antibody 3F4 as the anti-core antibody to macaque and human PrP.

**Figure 3:**
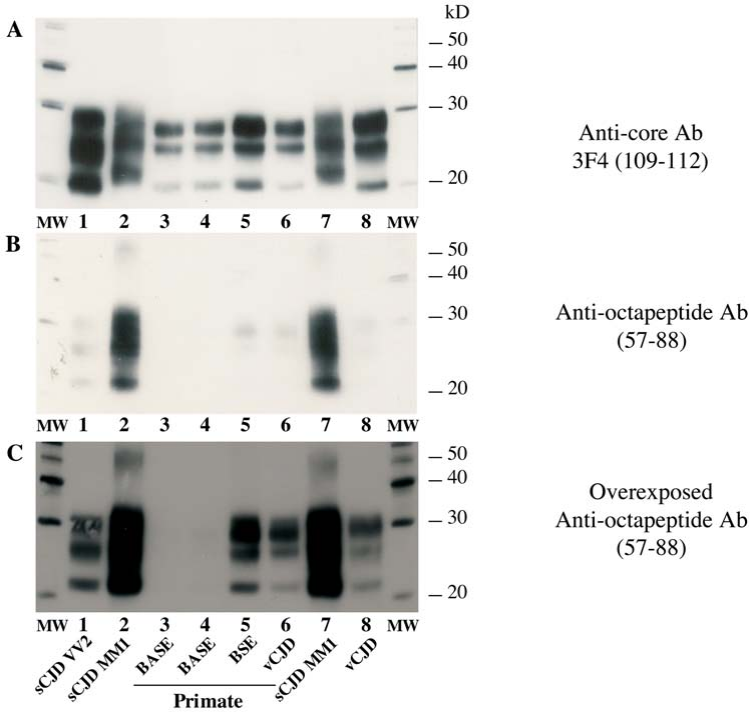
Electrophoretic analysis and differential sensitivity to proteolysis of PrPres in various prion diseases of primates and humans. PrPres from brain homogenates (MM1 or VV2 sCJD, vCJD in humans, or primates experimentally infected with BASE, cBSE or vCJD inocula) were purified under high concentrations of proteinase K, and detected with monoclonal antibodies recognizing either the core (3F4, panel A) or the octapeptide region (panel B and C) of the protein. Frontal cortex and obex regions of BASE-infected primate were both analysed (lanes 3 and 4 respectively). Panel C is an overexposure of the autoradiography of Panel B to detect weak signals. The absence of octarepeat region reactivity in the PrPres of the BASE in Panel C indicates a proportion at least ten fold lower than that of the vCJD or cBSE samples on the basis of a quantitative analysis of chemiluminescence signal intensities.

Banding patterns in Western blots following pre-treatment with a high PK concentration are shown in Figure 3. Both vCJD/cBSE and BASE reacted strongly to anti-core antibody (Panel A). In contrast, vCJD/cBSE also reacted weakly to anti-octapeptide antibody (Panel B), whereas BASE reactivity was abolished (Panels B and C), indicating a gradient of resistance to proteolysis of the N terminal part of the PrPres among these strains. In cattle, the signal was abolished for both cBSE and BASE strains (data not shown).

**Figure 4:**
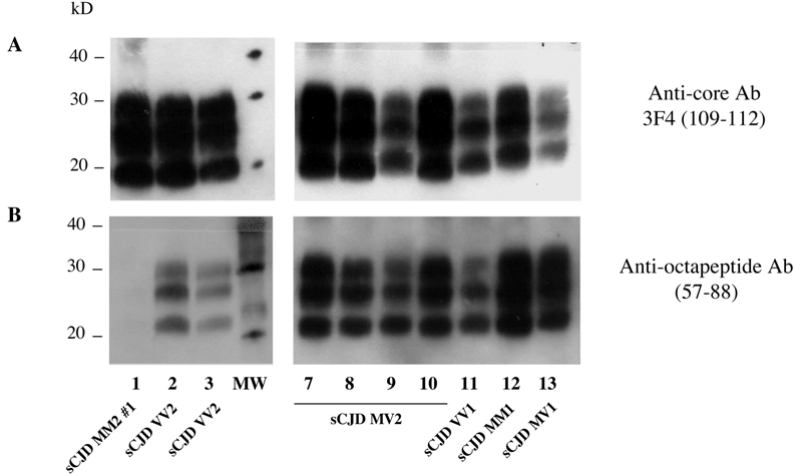
Electrophoretic analysis and differential sensitivity to proteolysis of PrPres in different subtypes of CJD. PrPres from human brain homogenates (MM1, MV1, VV1, MM2, MV2 or VV2 subtypes of sCJD, and vCJD) were purified under high concentrations of proteinase K, and detected with monoclonal antibodies that recognize either the core (3F4, Panel A) or the octapeptide region (Panel B) of the protein. The proportion of PrPres with an intact octarepeat region after PK exposure in VV2 and human (or macaque) vCJD was estimated to be only one-tenth and one-twentieth as high as in an MM1 sub-type of sCJD, respectively.

**Figure 5:**
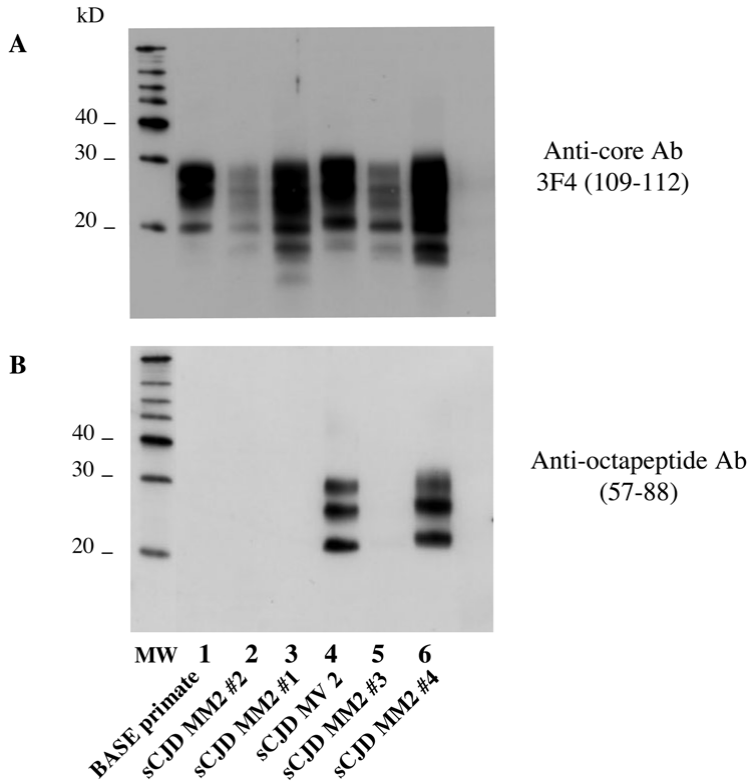
Electrophoretic analysis and differential sensitivity to proteolysis of PrPres in different MM2 CJD patients. PrPres from human brain homogenates (MM2 or MV2 subtypes of sCJD) and from primate experimentally infected with BASE were purified under high concentrations of proteinase K, and detected with monoclonal antibodies that recognize either the core (3F4, Panel A) or the octapeptide region (Panel B) of the protein.

The method also revealed notable differences of octapeptide sensitivity to PK in different types of human prion disease (figures 3 and 4). Comparisons of the relative signals with both anti-core and anti-octapeptide antibodies for each sample indicated that the N terminal part of PrPres from vCJD and the VV2 subtype of sCJD were far more sensitive than either the MM1 or VV1 subtypes (Panel B). The MV2 subtype showed a strong resistance to proteolysis that was clearly different from the BASE-infected primate; however, three of the four MM2 subtype cases exhibited the same signature as BASE, and the fourth case had a significant proportion of PrPres with an intact octapeptide region, as shown in figure 5, indicating the coexistence of two types of PrPres (the majority being type 2).

All four cases had clinical features consistent with the MM2 subtype as described by Gambetti et al [16]: comparatively long illnesses dominated by cognitive impairment followed by aphasia, and later in the course of illness the appearance of pyramidal and extrapyramidal signs together with myoclonus, but no cerebellar signs. Neuropathology was also typical of the MM2 subtype, with major cortical spongiosis and little or no involvement of the cerebellum (Table 2 summarizes the clinical, laboratory, and neuropathological features of each case).

**Table 2:** Summary of MM2 subtype sporadic CJD patients.

Case #	1	2	3	4
Sex	Female	Male	Male	Female
Age at onset	60 years	59 years	59 years	69 years
Duration of illness	24 months	9 months	6 months	16 months
Onset	Progressive memory impairment beginning with episodic memory disturbance, then attention loss, spatial and temporal orientation	Depression, insomnia, headache. Episodic memory impairment and poor language	Memory disturbance	Depression, memory impairment
Evolution	Progressive cognitive decline, motor and sensory aphasia, progressive global impairment of all cortical functions, with assistance needed for self care. Pyramidal signs mainly characterized by hypereflexia	Worsening of memory impairment, motor aphasia, intellectual decline, progressive loss of all cortical functions. Myoclonus, urinary incontinence, loss of self-care	Confusional state, global impairment of higher cortical functions	Progressive cognitive decline, characterized by global impairment of all cortical functions. Myoclonus
Terminal stage	Diffuse spastic rigidity; Pyramidal signs; dystonic movements and sporadic myoclonic jerks	Akinetic mutism	No information available	Akinetic mutism
MRI	Cortical atrophy with hyper-intensity of the head of Caudate nucleus and Putamen, in T2-weighted images	Normal (early stage of illness)	Normal (early stage of illness)	Hyperintensity in T2 weighted images of fronto-temporal cortical ribbon
SPECT 99mTc-ECD	Not done	Hypo-perfusion of frontal, parietal and temporal cortices, bilaterally. Normal perfusion of subcortical ganglia and cerebellum	Hypo-perfusion of frontal, parietal and to a lesser extent temporal cortices	Not Done
MMSE	10/30	Not done	Not done	Not reported
CSF 14-3-3 protein	Positive	Positive	Not done	Negative
EEG	Diffuse slowing.	Diffuse slowing	Diffuse slowing	Periodic sharp waves
Neuropathology	Cortical spongiosis, Cerebellum relatively spared	Cortical spongiosis, Cerebellum relatively spared	Cortical spongiosis, normal cerebellum	Cortical spongiosis, cerebellum relatively spared
Type PrPres	Type 2	Type 2	Type 2	Type 1+2
Resistance of N-terminal part to proteolysis	No (BASE infected primate-like)	No (BASE infected primate-like)	No (BASE infected primate-like)	Yes

## Discussion

We have shown that BASE, the first identified atypical strain of BSE [2], originating from asymptomatic cattle, is transmissible by i.c. inoculation to a species of non-human primate. Although this observation concerned only one animal, its survival was substantially shorter than for all the macaques inoculated with classical BSE as well as the majority of those inoculated with human vCJD. Moreover, in earlier experiments by others on a total of 6 macaques inoculated i.c. with 50 mg of cBSE brain, none had an incubation period of less than 30 months [17], and humanized transgenic mice have been found to be highly susceptible to infection with BASE, and completely resistant to infection with cBSE [18]. If BASE is more pathogenic than classical BSE for primates, it could indicate a more readily transmissible infection from cattle to humans than previously suspected. A preliminary trial of oral transmission is currently ongoing for alimentary risk assessment: 49 months after oral dosing there is no indication of transmission; however, the incubation period following similar oral challenge with cBSE in an already completed experiment was 60 months.

The disease induced by BASE was different in all respects from that induced by classical BSE. The clinical presentation was characterized by mild tremors and myoclonus, progressing to a marked cognitive disorder, including spatial disorientation but without anxiety, aggressiveness or loss of appetite. In contrast, cBSE presented signs of anxiety and aggressiveness together with progressive difficulties in locomotion as well as cerebellar signs (major ataxia), and severe decrease of appetite with concurrent weight loss. The widespread spongiform lesions and loss of pyramidal cells in the third and fifth layers of the frontal cortex together with the severe parietal lesions could explain the prominent cognitive signs and the spatial disorientation seen in the BASE-infected monkey, contrasting with the severity of lesions in the obex and cerebellum consistent with the incoordination seen in animals inoculated with cBSE. Amyloid plaques, the hallmark of BASE in cattle, are not produced in the Macaque monkey, and conversely, cBSE does not produce plaques in cattle, but does so in the Macaque [9], a clear indication that plaque deposition depends as much on the host as the prion strain.

At the molecular level, under conditions of high proteinase pre-treatment and detection using two antibodies reacting with either an epitope in the N terminal octapeptide repeat region or the core of PrP, BASE and cBSE were clearly distinguishable in primate. BASE was detectable only by the core antibody, whereas cBSE was detectable by both antibodies. We estimated that the proportion of octapeptide-resistant PrPres molecules in the BASE brain homogenate was only a small fraction (≤1/10) of that of the cBSE brain homogenate. The difference in octapeptide sensitivity to PK between cBSE and vCJD in macaques on the one hand, and Type 1 sporadic CJD in humans on the other hand, is similar to what was observed between cBSE and classical scrapie in sheep. This method can now be used to test both ruminant and human samples to identify similarities and differences in their molecular protein signatures, and to implement the classification of ruminant and possibly human strains.

Although classical epidemiological studies have not found any link between scrapie in sheep and goats and human CJD, newer molecular biological studies now indicate that about half of all cases of scrapie are due to previously undetected atypical strains [19] that are experimentally transmissible to sheep and mice [20]. Their risk for humans is unknown and is the subject of current studies in experimental models, including primates. cBSE has been shown to be responsible for human cases of vCJD, but the comparative risk for humans of BASE and other atypical strains of BSE is still unknown, and its clarification will require many years of epidemiological surveillance and molecular biological testing of both bovine and human populations.

The first cases of BASE in cattle had PrPres electrophoretic profiles similar to the MV2 subtype of sporadic CJD patients [2] that, together with the presence of amyloid plaques in both the cattle and the patients, suggested a possible link between BASE and this subtype of sCJD. However, our PrPres typing technique has shown that, in the primate, PrPres of other MV2 sCJD patients exhibited a resistance to proteolysis different from the BASE-infected primate, whereas PrPres from vCJD-infected patients and primates behave similarly. This observation, together with the absence of amyloid plaques in the BASE-infected primate, weakens the likelihood of a direct link between BASE and MV2 subtype sCJD patients.

In contrast, the specific signature of PrPres in the BASE-infected primate was similar to that seen in three of four patients with the MM2 cortical subtype of sporadic CJD [7]. It is interesting that an important feature of the clinical-pathological syndrome in this BASE-infected macaque –the absence of cerebellar involvement – is also a common element in patients with the MM2 subtype of human sporadic CJD (Supplementary Figure S2). However, as illustrated by the clinical details of our four tested MM2 cases, there is considerable patient-to-patient variation, just as there can be variation among individual animals experimentally inoculated with a given strain of TSE. [21], [22].

It is not known whether atypical strains of BSE have been circulating for years, or represent new forms of disease, and continuing research is clearly needed to answer both this and the equally important question about a possible relationship to at least certain forms of what are presently regarded as sporadic cases of human disease (sCJD) [4], [23]. Moreover, the BASE strain has been described to evolve naturally towards BSE after successive transmissions in inbred mice [6]. The stability and pathogenicity of this strain in humans remains to be determined, and it is worth recalling that the stability of the cBSE/vCJD strain, which retains its specific molecular signature in different infected hosts, is the exception rather than the rule. As has been previously observed [24]–[26], one patient (Case No. 4, cf. figure 5 sample MM2#4) exhibited both types of PrP, i.e. type 2 typical of the MM2 subtype and type 1 observed in the MM1 subtype. On the one hand, this demonstrates the interest of such a simple biochemical test to refine PrP analysis, and on the other hand it raises a question about the existence of different PrPres signatures in the same patient, i.e., different prion strains linked to multiple infections or to variants selected by the host.

In summary, we have transmitted one atypical form of BSE (BASE) to a cynomolgus macaque monkey that had a shorter incubation period than monkeys infected with classical BSE, with distinctive clinical, neuropathological, and biochemical features; and have shown that the molecular biological signature resembled that seen in a comparatively uncommon subtype of sporadic CJD. We cannot yet say whether BASE is more pathogenic for primates (including humans) than cBSE, nor can we predict whether its molecular biological features represent a clue to one cause of apparently sporadic human CJD. However, the evidence presented here and by others justifies concern about a potential human health hazard from undetected atypical forms of BSE, and despite the waning epizoonosis of classical BSE, it would be premature to abandon the precautionary measures that have been so successful in reversing the impact of cBSE. We would instead urge a gradual, staged reduction that takes into account the evolving knowledge about atypical ruminant diseases, and both a permanent ban on the use of bovine central nervous system tissue for either animal or human use, and its destruction so as to eliminate any risk of environmental contamination.

## Supporting Information

Figure S1 Resistance to proteolysis of different prion strains in sheep. PrPres from brain homogenates of sheep infected with classical scrapie, experimental cBSE, or atypical Nor-98 scrapie, and of an uninfected control sheep. Samples were purified using low (odd lanes) or high (even lanes) concentrations of proteinase K, and visualized with monoclonal antibodies that recognize either the core region (Panel A) or the octapeptide region (Panel B) of the protein. With the lower concentration of PK used in the purification step (in order to maximize test sensitivity) of one widely utilized BSE screening test [13], all three strains gave a positive result with both the anti-core and anti-octapeptide antibodies (odd lanes). Using a higher concentration of PK (even lanes) did not alter the positivity with either antibody for classical scrapie, but the cBSE strain no longer reacted with the anti-octapeptide antibody while Nor98 did not react with either antibody. Thus, by using the higher concentration of PK and two different antibodies, it is possible to discriminate between all three strains.
(2.51 MB TIF)

Figure S2 Lesion profiles in cBSE- and BASE-infected macaque, and in MM2 sporadic CJD patients. The lesions were scored from 0 to 4 (negative, light, mild, moderate, and severe) for the different following gray matter regions: frontal (FC), temporal (TC), parietal (PC) and occipital (OC) neocortices, hippocampus (HI), parasubiculum and entorhinal cortex (EC), neostriatum (ST) (nuclei caudatus and putamen), thalamus (TH), substantia nigra (SN), midbrain periventricular gray (PG), locus ceruleus (LC), medulla (ME) (periventricular gray and inferior olive) and cerebellum (CE). Scoring for MME sCJD patient was issued from Parchi et al. [7].
(1.52 MB TIF)
