# Supplementary figures and images for: Atypical BSE (BASE) Transmitted from Asymptomatic Aging Cattle to a Primate
Source: PLoS One. 2008 Aug 20;3(8):e3017. doi: 10.1371/journal.pone.0003017 (PMC2515088; doi:10.1371/journal.pone.0003017)

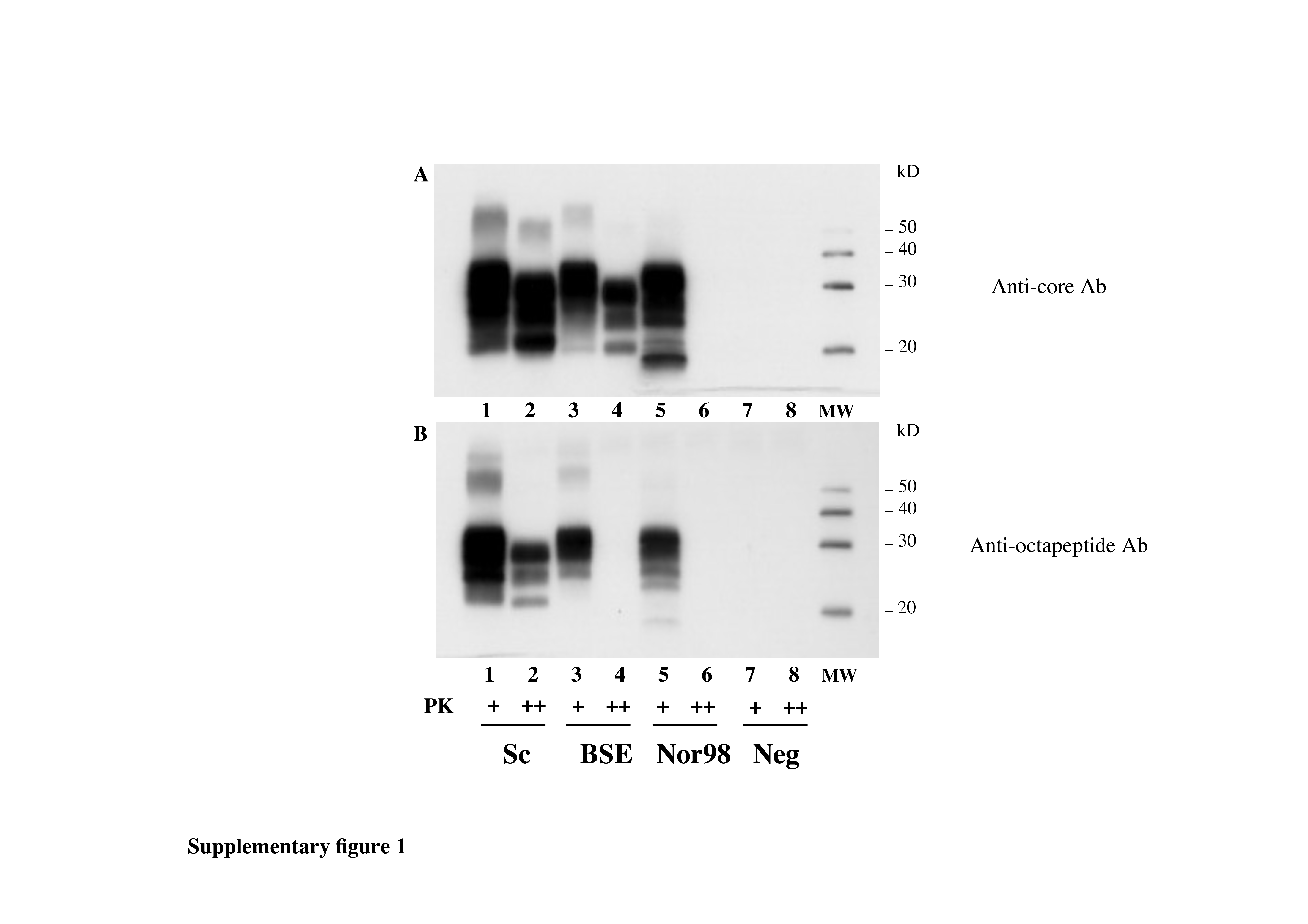

Supplement: Figure S1 — Resistance to proteolysis of different prion strains in sheep. PrPres from brain homogenates of sheep infected with classical scrapie, experimental cBSE, or atypical Nor-98 scrapie, and of an uninfected control sheep. Samples were purified using low (odd lanes) or high (even lanes) concentrations of proteinase K, and visualized with monoclonal antibodies that recognize either the core region (Panel A) or the octapeptide region (Panel B) of the protein. With the lower concentration of PK used in the purification step (in order to maximize test sensitivity) of one widely utilized BSE screening test [13], all three strains gave a positive result with both the anti-core and anti-octapeptide antibodies (odd lanes). Using a higher concentration of PK (even lanes) did not alter the positivity with either antibody for classical scrapie, but the cBSE strain no longer reacted with the anti-octapeptide antibody while Nor98 did not react with either antibody. Thus, by using the higher concentration of PK and two different antibodies, it is possible to discriminate between all three strains. (2.51 MB TIF) [file pone.0003017.s001.tif]

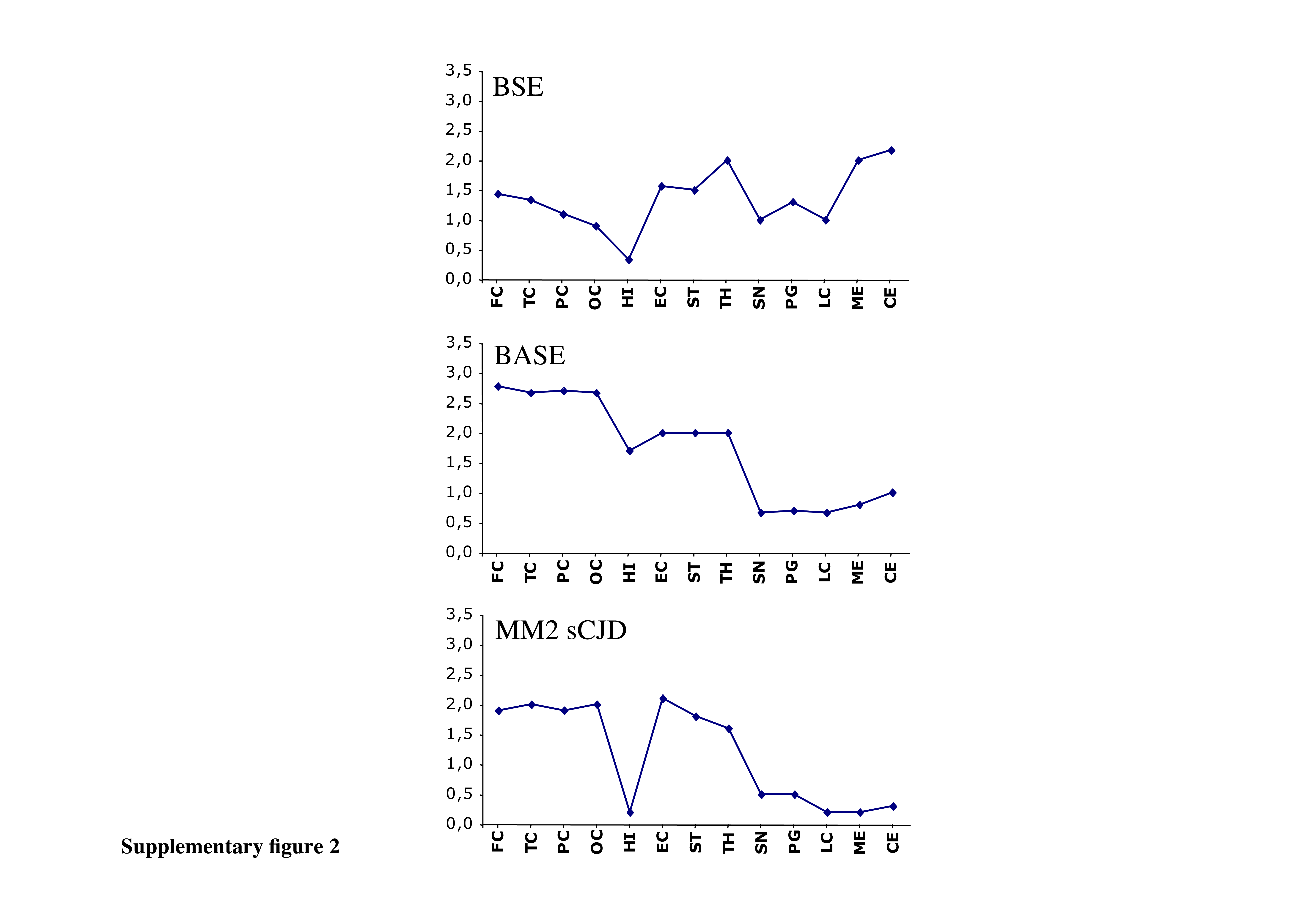

Supplement: Figure S2 — Lesion profiles in cBSE- and BASE-infected macaque, and in MM2 sporadic CJD patients. The lesions were scored from 0 to 4 (negative, light, mild, moderate, and severe) for the different following gray matter regions: frontal (FC), temporal (TC), parietal (PC) and occipital (OC) neocortices, hippocampus (HI), parasubiculum and entorhinal cortex (EC), neostriatum (ST) (nuclei caudatus and putamen), thalamus (TH), substantia nigra (SN), midbrain periventricular gray (PG), locus ceruleus (LC), medulla (ME) (periventricular gray and inferior olive) and cerebellum (CE). Scoring for MME sCJD patient was issued from Parchi et al. [7]. (1.52 MB TIF) [file pone.0003017.s002.tif]
